# Supplementary material for: Cardiopulmonary Ultrasound Patterns of Transient Acute Respiratory Distress of the Newborn: A Retrospective Pilot Study
Source: Children (Basel). 2023 Feb 2;10(2):289. doi: 10.3390/children10020289 (PMC9955778; doi:10.3390/children10020289)
Supplement: Supplementary file 1 [file children-10-00289-s001.zip › children-2121723-supplementary.pdf]

children

MDPI

Article

Cardiopulmonary ultrasound patterns of transient acute respiratory distress of the newborn: a retrospective pilot study

Maria Pierro [1,*], Roberto Chioma [2], Consuelo Benincasa [1,3], Giacomo Gagliardi [1,3], Lorenzo Amabili [4], Francesca Lelli [1,3], Patrizio Antonazzo [5], Giovanni De Luca [6] and Enrico Storti [7]

1 Neonatal and Paediatric Intensive Care Unit, M. Bufalini Hospital, AUSL Romagna, Cesena, Italy;
2 Neonatal Intensive Care Unit, Catholic University of Sacred Heart, Rome, Italy.
3 Paediatric Unit, Ferrara University Hospital, Ferrara, Italy;
4 Mosaic factor, Barcelona, Spain
5 Unit of Obstetrics and Gynecology, Bufalini Hospital-AUSL Romagna, Cesena, Italy
6 Pathology and Oncohematology department, M. Bufalini Hospital, AUSL Romagna, Cesena, Italy
7 Department of Critical Care, Maggiore Hospital, Cremona, Italy.
* Correspondence: Maria Pierro; maria.pierro93gmail.com; maria.pierro@auslromagna.it; Neonatal and Paediatric Intensive Care Unit, M. Bufalini Hospital, AUSL Romagna, Viale G. Ghirotti, 286 - 47521, Cesena, Italy; Phone number: +39/0547352844

*Correspondence: Maria Pierro
maria.pierro93gmail.com; maria.pierro@auslromagna.it
Neonatal and Paediatric Intensive Care Unit
M. Bufalini Hospital,AUSL Romagna
Viale G. Ghirotti, 286 - 47521, Cesena, Italy
Phone number: +39/0547352844

SUPPLEMENTAL MATERIAL

MATERIAL AND METHODS

Definitions of the prenatal collected data are as follows:

- diabetes in pregnancy as defined by NICE guidelines in presence of a fasting plasma glucose level of 5.6 mmol/liter or above or a 2-hour plasma glucose level of 7.8 mmol/liter or above [1], or preexisting diabetes [2]. Type of diabetes was recorded

- Gestational disorders of pregnancy were defined according to the American College of Obstetricians and Gynecologists (ACOG) guidelines [3]: preexisting or gestational hypertension, defined by the new onset of hypertension (systolic blood pressure ≥140 mmHg and/or diastolic blood pressure ≥90 mmHg) at ≥20 weeks of gestation in the absence of proteinuria or new signs of end-organ dysfunction, preeclampsia refers to the new onset of hypertension and proteinuria or the new onset of hypertension and significant end-organ dysfunction with or without proteinuria after 20 weeks of gestation or postpartum in a previously normotensive patient, HELLP syndrome (hemolysis with a microangiopathic blood smear, elevated liver enzymes, and a low platelet count); chronic hypertension (systolic blood pressure ≥140 mmHg and/or diastolic blood pressure ≥90 mmHg) before 20 weeks gestation

- "Intrauterine Inflammation or Infection or both" also known as "Triple-I syndrome" was defined as the presence of intrapartum fever with at least one of the following items: fetal tachycardia (> 160 bpm for more than 10 minutes), maternal WBC > 15,000 in absence of corticosteroid treatment, purulent fluid from the cervical canal and bio-chemical or microbiologic amniotic fluid results consistent with microbial invasion of the amniotic cavity. Instead, a clinically documented fever with a temperature measured orally higher than 39.0°C or 102.2°F once or 38.0°C or 100.4°F twice was defined as isolated maternal fever [4].

- Intrauterine growth restriction (IUGR) was defined as a weight below the 10th percentile at fetal ultrasound [5].

- Fetal heart monitoring: intrapartum fetal heart health monitoring with non-invasive electronic monitoring classified in three categories: category 1 (normal) when fetal heart rate (FHR) baseline is between 110 and 160 bpm with moderate FHR variability (6 to 25 bpm) and absence of late, variable, or prolonged FHR decelerations; category 3 (pathological) if there are sinusoidal pattern, absent baseline FHR variability or recurrent late decelerations, recurrent variable decelerations, bradycardia; category 2 (indeterminate) FHR tracing does not meet criteria for either category I or III [6].

Placental histology

We retrospectively evaluated also placental histology. The presence of maternal vascular malperfusion (MVM) and chorioamnionitis on the placental histologies was defined according to the Amsterdam International Consensus criteria [7], and examined under the conventional microscope after hematoxylin and eosin staining. The placental pathologic findings involving maternal decidual vessels, reflecting abnormal spiral artery remodeling, and villous parenchyma, reflecting abnormalities in oxygenation and flow dynamics in the intervillous space, are summarized by the term MVM of the placental bed. MVM was defined by both macroscopic and microcopic placental findings. Macroscopic features include placental hypoplasia (with a weight and/or a thin cord

below 10th centile per gestational age), infarction and retroplacental hemorrhage. Microscopic findings include abnormalities of villous development, divided into distal villous hypoplasia and accelerated villous maturation. In case of chorioamnionitis (CA), we described the staging and grading of inflammation formulated in the Amsterdam International Consensus criteria. Preacute chorioamnionitis (stage 0) is characterised by neutrophils in the space beneath the chorionic plate. Early chorioamnionitis (stage 1) is defined by neutrophils limited to the chorion (chorionitis). Intermediate (stage 2) is distinguished by neutrophils within the chorionic or amniotic mesoderm.

Advanced chorioamnionitis (stage 3) included inflammation of the chorioamniotic membreanes with necrosis of amniotic epithelium or neutrophil necrosis. About grading we described severe if there were confluent polymorphonuclear leukocytes or with subchorionic

FIGURES

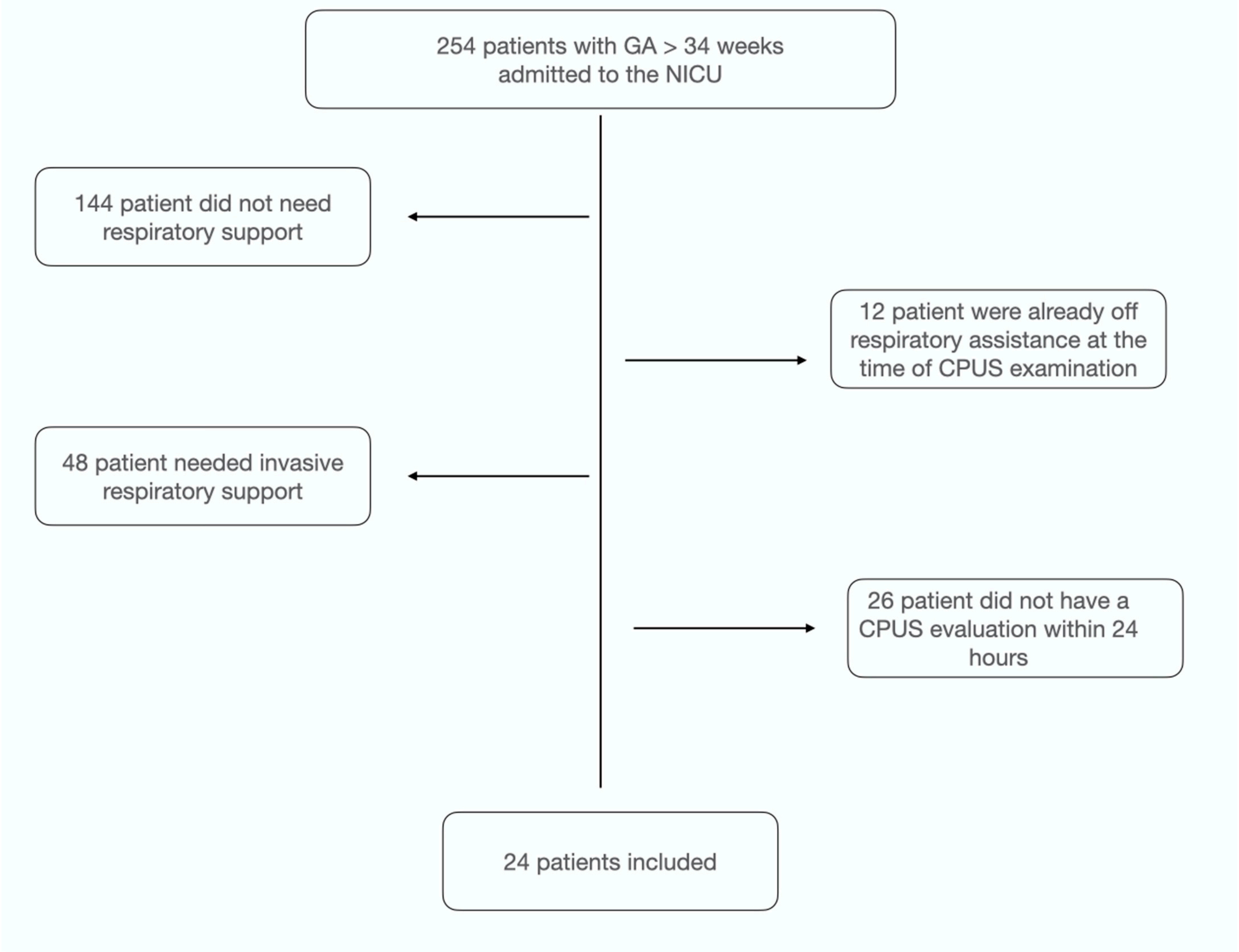

Figure S1. Patient population flow chart

Abbreviations: gestational age (GA), neonatal intensive care unit (NICU), cardiopulmonary ultrasound (CPUS)

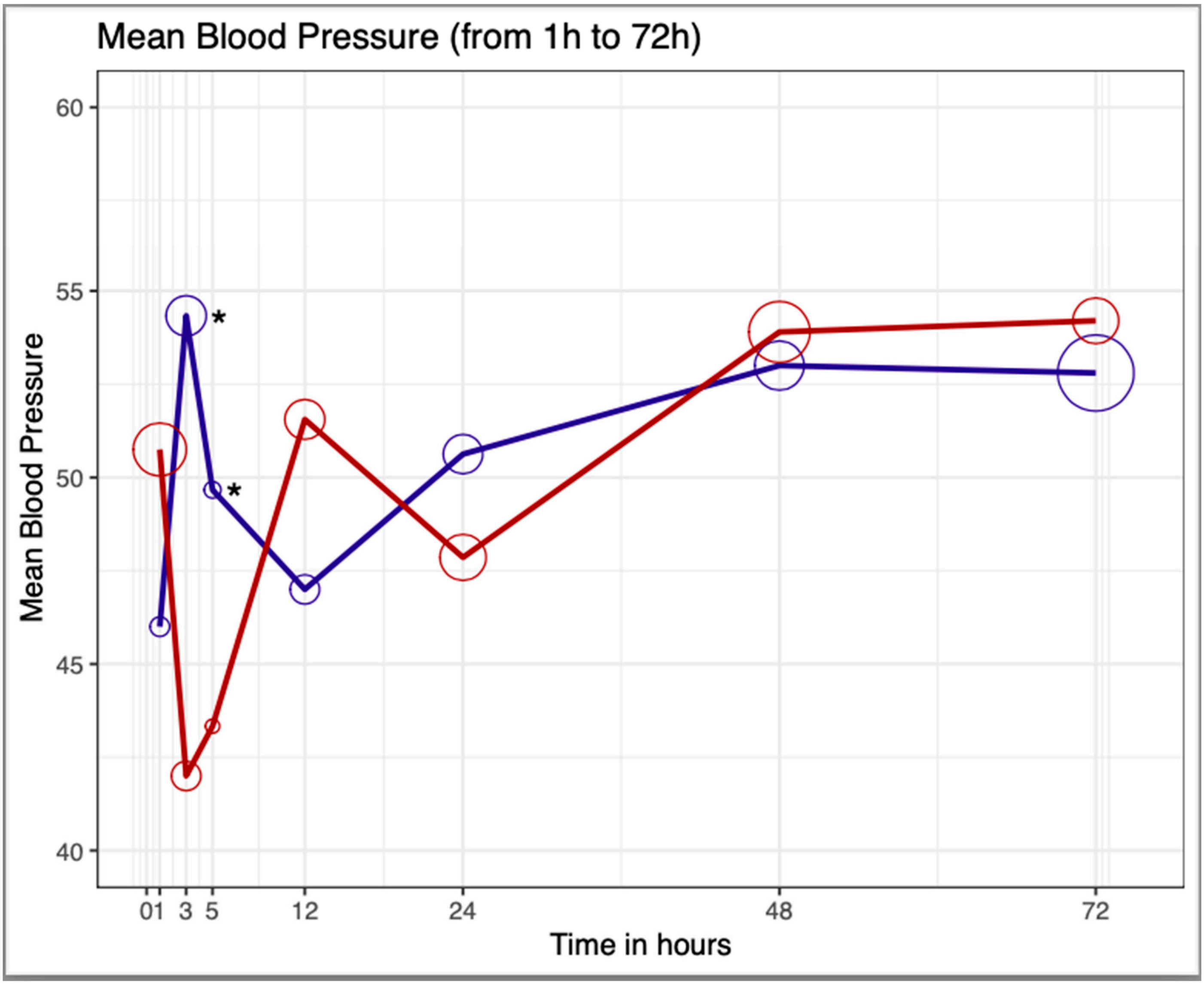

Figure S2 Blood pressure trends.
Blood pressure trends in infants with increased pulmonary vascular resistances (red line) and normal vascular resistances (blue line). Circles represent standard deviations

TABLES

Table S1 - Charateristics of the Acute Respiratory Distress Phenotype Subgroups

**Characteristics**	**Undefined (AND-U) (n=2)**	**Vascular (AND-v) (n=4)**	**Wet lung (AND-w) (n=4)**	**Vascular-wet lung (AND-vw) (n=4)**	**Vascular-RDS (n=2)**	**Consolidation (AND-p) (n=6)**	**Vascular-consolidation (AND-vc)** (n=2)	**p-value**
Maternal diabetes, n (%)	2 (100%)	0	1 (25%)	1 (25%)	0	0	0	0.628
Triple -I, n (%)	0	1 (25%)	3 (75%)	1 (25%)	1 (50%)	1 (16.7%)	1 (50%)	0.466
Placenta previa, n (%)	0	1 (25%)	0	0	0	1 (16.7%)	0	0.802
Fetal tachicardia, n (%)	1 (50%)	1 (25%)	1 (33.3%)	0	1 (50%)	2 (33.3%)	0	0.990
Maternal tachicardia, n (%)	0	0	0	0	0	1 (16.7%)	0	0.909
Maternal fever, n (%)	0	1 (25%)	0	1 (25%)	0	1 (16.7%)	0	0.925
Intrapartum antibiotics, n (%)	0	1 (25%)	2 (66.7%)	2 (50%)	2 (100%)	1 (16.7%)	0	0.241
Chorioamnionitis								0.478
No, n (%)	1 (50%)	2 (50%)	3 (75%)	1 (50%)	1 (50%)	5 (83.3%)	0	
Histological, n (%)	1 (50%)	1 (25%)	1 (25%)	1 (50%)	1 (50%)	0	2 (100%)	
Clinic, n (%)	0	1 (25%)	0	0	0	1 (16.7%)	0	
Placenta malperfusion, n (%)	1 (50%)	3 (75%)	1 (25%)	1 (25%)	2 (100%)	2 (33.3%)	2 (100%)	0.264
Gestational age (weeks), median (IQR)	40.4 (39.7-41.1)	41 (35.8-41.8)	37.5 (36-40.3)	36.8 (36-40.3)	38 (35.7-39.3)	40.2 (39-41)	40.3 (39-41.6)	0.729
Birth weight (grams), median (IQR)	3700 (3500-3900)	3400 (2720-3690)	3590 (3030-4388)	2800 (2710-3200)	3200 (2100-4300)	3400 (3080-3540)	3500 (3200-3800)	0.453
Need for any kind of resuscitation at birth, n (%)	1 (50%)	3 (75%)	0	0	1 (50%)	4 (66.7%)	2 (100%)	0.072
Apgar at 1 minute, median (IQR)	7.5 (6-9)	5 (3.5-8)	9 (9-9)	9 (9-9)	6.5 (4-9)	7 (5-9)	5.5 (5-6)	0.153
Apgar at 5 minutes, median (IQR)	9 (8-10)	7.5 (6-9)	10 (10-10)	10 (10-10)	7.5 (5-10)	8 (7-9)	7.5 (7-8)	0.061
Amniotic fluid								0.126
Clear, n (%)	2 (100%)	1 (25%)	3 (100%)	4 (100%)	1 (100%)	1 (16.7%)	0	
Meconium stained, n (%)	0	3 (75%)	0	0	0	4 (66.7%)	2 (100%)	
Blood stained, n (%)	0	0	0	0	0	1 (16.7%)	0	

Maximum CRP (mg/dL) , median (IQR)	0.9 (0.9-0.9)	3 (0.5-5.4)	5.2 (1.6-6.5)	2 (1.4-3.5)	18.5 (5-30)	1.2 (0-2)	3.4 (3.-3.8)	0.001
Positive blood culture, n (%)	0	0	0	0	1 (50%)	0	0	0.550
Antibiotic therapy, n (%)	0	3 (75%)	3 (75%)	0	2 (100%)	1 (16.7%)	1 (50%)	0.055
Antibiotics duration (days), median (IQR)	0 (0-0)	3.5 (0.7-4)	3 (0.5-4.7)	0 (0-0)	8.5 (2-15)	0 (0-2)	1.5 (0-3)	
Length of stay (day), median (IQR)	11.5 (5-18)	8 (6-12)	9 (7-11)	4 (3-5)	14.5 (11-18)	4 (3.5-8)	6.5 (6-7)	0.094
Duration of CPAP (hours), median (IQR)	15 (0-30)	16 (0-60)	65 (13-78)	12 (0-60)	80 (58-102)	24 (8-32)	27 (5-49)	0.211
Duration of HFNC (hours), median (IQR)	115 (0-230)	3 (0-56)	1.5 (0-41.2)	0 (0-36)	6.5 (1-12)	0 (0-21)	0 (0-0)	0.266
Duration of NRS/low-flow oxygen (hours), median (IQR)	133 (30-236)	54 (8-92)	70 (55-84)	38 (25-66)	96.5 (88-105)	15.5 (4-59)	27 (5-49)	0.207
Maximum FiO2, median (IQR)	0.35 (0.30-0.40)	0.30 (0.30-0.38)	0.40 (0.26-0.55)	0.25 (0.30-0.35)	0.50 (0.50-0.50)	0.30 (0.26-0.45)	0.40 (0.40-0.40)	0.752
Surfactant administration via INSURE, n (%)	0	1 (25%)	1 (25%)	0	1 (50%)	1 (16.7%)	0	0.731
Hours of life at CPUS, median (IQR)	18 (10-22)	18 (10-21)	14 (6-22)	9 (5-19)	23.5 (23-24)	15 (5-26)	13 (2-24)	0.692
FiO2 at CPUS, median (IQR)	0.33 (0.27-0.39)	0.28 (0.28-0.38)	0.35 (0.24-0.50)	0.25 (0.30-0.35)	0.45 (0.40-0.50)	0.30 (0.24-0-40)	0.38 (0.36-0.40)	0.354

Abbreviations: Intrauterine growth restriction (IUGR), non invasive respiratory support (NRS), continuous positive airway pressure (CPAP), high flow nasal cannula (HFNC), C-reactive protein (CRP)

Table S2 - Charateristics of the **Vascular/not Vascular** Phenotype Subgroups

**Characteristics**	**Normal PVR (n=12)**	**Increased PVR (n=12)**	**p-value**
Maternal diabetes, n (%)	1 (8.3%)	1 (8.3%)	1
Triple I, n (%)	4 (33.3%)	4 (33.3%)	1
Fetal tachicardia, n (%)	4 (36.4%)	2 (16.7%)	0.549
Maternal tachicardia, n (%)	1 (11.1%)	0	1
Maternal fever, n (%)	1 (10%)	2 (18.2%)	1
Intrapartum antibiotics, n (%)	3 (30%)	5 (41.7%)	0.903
Chorioamnionitis			0.140
No, n (%)	9 (75%)	4 (33.3%)	
Histology, n (%)	2 (16.7%)	6 (55.6%)	
Clinic, n (%)	1 (8.3%)	2 (11.1%)	
Placenta malperfusion, n (%)	4 (33.3%)	8 (66.7%)	0.071
Gestational age (weeks), median (IQR)	40 (37.2-41)	39.5 (35.8-42.5)	0.932
Birth weight (grams), median (IQR)	3480 (3300-3740)	3230 (2700-3700)	0.160
Need for any kind of resuscitation at birth, n (%)	5 (41.7%)	6 (50%)	1
Apgar at 1 minute, median (IQR)	9 (6.2-9)	6 (5-9)	0.347
Apgar at 5 minutes, median (IQR)	9.5 (8-10)	8 (7-10)	0.347
Amniotic fluid			0.574
Clear, n (%)	6 (54.4%)	6 (50%)	
Meconium stained, n (%)	5 (36.4%)	6 (50%)	
Blood stained, n (%)	1 (9.1%)	0	
Maximum CRP (mg/dL), median [IQR]	1.8 (0.7-5)	2.8 (1.4-4.7)	0.264
Positive blood culture, n (%)	0	1 (16.7%)	1
Antibiotic therapy, n (%)	4 (33.3%)	6 (50%)	0.679
Antibiotics duration (days), median (IQR)	0 (0-3.5)	1 (0-3.8)	0.59
Length of stay (days), median (IQR)	7 (4-11)	6 (6-11)	0.746
Duration of CPAP (hours), mean (IQR)	28.5 (3-51)	28 (0-67)	0.775

Duration of HFNC (hours), mean (IQR)	0 (0-41)	0 (0-10.5)	0.367
Duration of NRS/low-flow oxygen (hours), median (IQR)	48 (13-85)	49 (25-85)	0.692
Maximum FiO2, median (IQR)	0.35 (0.29-0.40)	0.35 (0.30-0.40)	0.875
Surfactant administration via INSURE, n (%)	2 (16.7%)	2 (16.7%)	1
Hours of life at CPUS, median (IQR)	15 (7.2-23.5)	18 (6.2-22.5)	0.977
FiO2 at CPUS, median (IQR)	0.33 (0.28-0.40)	0.34 (0.28-0.38)	0.833

Abbreviations: Intrauterine growth restriction (IUGR), non invasive respiratory support (NRS), continuous positive airway pressure (CPAP), high flow nasal cannula (HFNC), C-reactive protein (CRP)

Table S3 - Characteristics of the lung phenotype groups

**Characteristics**	**Normal**<br>**n=6**	**Wet lung**<br>**n=8**	**RDS**<br>**n=2**	**Consolidation**<br>**n=8**	***p*-value**
Maternal diabetes, n (%)	0	0	2 (28.6%)	0	0.23
Triple I, n (%)	1 (16.7%)	4 (100%)*	1 (14.3%)*	2 (28.6%)	0.03
Placenta previa, n (%)	1 (16.7%)	0	0	1 (14.3%)	1
Fetal tachicardia, n (%)	2 (33.3%)	1 (25%)	1 (14.3%)	2 (28.6%)	0.92
Maternal tachicardia, n (%)	0	0	0	1 (14.3%)	1
Maternal fever, n (%)	1 (16.7%)	1 (25%)	0	1 (14.3%)	0.33
Intrapartum antibiotics, n (%)	3 (50%)	3 (75%)	2 (28.6%)	2 (28.6%)	0.45
Chorioamnionitis<br>No, n (%)<br>Histology, n (%)<br>Clinic, n (%)	 3 (50%)<br>2 (33.3%)<br>1 (16.7%)	 4 (50%)<br>4 (50%)<br>0	 1 (50%)<br>1 (50%)<br>0	 4 (57.1%)<br>3 (28.6%)<br>1 (14.3%)	1
Placenta malperfusion, n (%)	4 (66.7%)	2 (50%)	2 (28.6%)	4 (57.1%)	0.63
Gestational age (weeks), median (IQR)	40.7 (38.3-41)	37.5 (36-2-40.3)	37.6 (36-41)	40 (39-41)	0.524
Birth weight (grams), median (IQR)	3510 (3097-3787)	3590 (2860-4388)	2900 (2790-3550)	3380 (3170-3500)	0.654
Need for any kind of resuscitation at birth, n (%)	4 (66.7%)	0§	1 (14.3%)*	6 (75%)*§	0.01
Apgar at 1 minute, median (IQR)	5.5 (4.5-9)	9 (9-9)	9 (9-9)	6 (5-7)	0.058
Apgar at 5 minutes, median (IQR)	8 (6,7-9.2)*	10 (10-10)*†	10 (10-10)§	8 (7-8)†§	0.017
Amniotic fluid<br>Clear, n (%)<br>Meconium stained, n (%)<br>Blood stained, n (%)	 3 (50%)<br>3 (50%)<br>0	 4 (100%)§<br>0<br>0	 7 (100%)*§<br>0*<br>0	 0*<br>6 (85,7)*<br>1 (14.3%)	0.001
Maximum CRP (mg/dL), median (IQR)	1.5 (0.5-5)	5.2 (3.6-6.5)	1.7 (0.6-10)	1.92 (0.9-3)	0.722
Positive blood culture, n (%)	0	0	1 (50%)	0	0.813
Antibiotic therapy, n (%)	3 (50%)	3 (37%)	2 (100%)	2 (25%)	0.633
Antibiotics duration (days), median (IQR)	1.5 (0-4)	3 (0.5-4.7)	0 (0-2)	0 (0-3)	0.929
Length of stay (days), median (IQR)	8 (5.7-14.2)	8 (6.2-9.8)	7 (3.5-14)	4 (4-7)	0.415
Duration of CPAP (hours), median (IQR)	15 (0-41)	38 (0-78)	54 (0-72)	28 (11-41)	0.574
Duration of HFNC (hours), median (IQR)	3 (0-112)	1.5 (0-41.2)	0 (0-12)	0 (0-0)	0.440
Duration of NRS/low-flow oxygen (hours), median (IQR)	54 (22.5-132)	70 (35-84)	54 (24-88)	20 (5-50)	0.506
Maximum FiO2, median (IQR)	0.30 (0.30-0.40)	0.40 (0.32-0.55)	0.30 (0.21-0.50)	0.40 (0.30-0.40)	0.526
Surfactant administration via INSURE, n (%)	1 (16.7%)	1 (12.5%)	1 (50%)	1 (12.5%)	1
Hours of life at CPUS, median (IQR)	18 (10-22)	14 (7-22)	20 (5-24)	3 (13-24)	0.798
FiO2 at CPUS, median (IQR)	0.28 (0.27-0.36)	0.35 (0.32-0.50)	0.30 (0.21-0.45)	0.35 (0.38-0.40)	0.523

Abbreviations: Intrauterine growth restriction (IUGR), non invasive respiratory support (NRS), continuous positive airway pressure (CPAP), high flow nasal cannula (HFNC), C-reactive protein (CRP). *§: symbols represent significance among groups. Two groups with the same symbol are significant among each other

References

1. Walker, J. D. NICE guidance on diabetes in pregnancy: management of diabetes and its complications from preconception to the postnatal period. NICE clinical guideline 63. London, March 2008. Diabet Med 2008, 25 (9), 1025-7. DOI: 10.1111/j.1464-5491.2008.02532.x .
2. 2. Classification and Diagnosis of Diabetes: Standards of Medical Care in Diabetes-2020. Diabetes Care 2020, 43 (Suppl 1), S14-s31. DOI: 10.2337/dc20-S002 .
3. Gestational Hypertension and Preeclampsia: ACOG Practice Bulletin, Number 222. Obstet Gynecol 2020, 135 (6), e237-e260. DOI: 10.1097/aog.0000000000003891 .
4. Peng, C. C.; Chang, J. H.; Lin, H. Y.; Cheng, P. J.; Su, B. H. Intrauterine inflammation, infection, or both (Triple I): A new concept for chorioamnionitis. Pediatr Neonatol 2018, 59 (3), 231-237. DOI: 10.1016/j.pedneo.2017.09.001 .
5. Ott, W. J. Sonographic diagnosis of fetal growth restriction. Clin Obstet Gynecol 2006, 49 (2), 295-307. DOI: 10.1097/00003081-200606000-00011 .
6. Macones, G. A.; Hankins, G. D.; Spong, C. Y.; Hauth, J.; Moore, T. The 2008 National Institute of Child Health and Human Development workshop report on electronic fetal monitoring: update on definitions, interpretation, and research guidelines. Obstet Gynecol 2008, 112 (3), 661-6. DOI: 10.1097/AOG.0b013e3181841395 .
7. Khong, T. Y.; Mooney, E. E.; Ariel, I.; Balmus, N. C.; Boyd, T. K.; Brundler, M. A.; Derricott, H.; Evans, M. J.; Faye-Petersen, O. M.; Gillan, J. E.; Heazell, A. E.; Heller, D. S.; Jacques, S. M.; Keating, S.; Kelehan, P.; Maes, A.; McKay, E. M.; Morgan, T. K.; Nikkels, P. G.; Parks, W. T.; Redline, R. W.; Scheimberg, I.; Schoots, M. H.; Sebire, N. J.; Timmer, A.; Turowski, G.; van der Voorn, J. P.; van Lijnschoten, I.; Gordijn, S. J. Sampling and Definitions of Placental Lesions: Amsterdam Placental Workshop Group Consensus Statement. Arch Pathol Lab Med 2016, 140 (7), 698-713. DOI: 10.5858/arpa.2015-0225-CC .
